# A Rare Presentation of Renal Sarcoidosis with Severe, Recurrent Hypercalcemia Despite Low-normal Vitamin D Levels

**DOI:** 10.1210/jcemcr/luaf255

**Published:** 2025-10-30

**Authors:** Reshma Reguram, Carly Hubers, Kendall Conway, Sudhanva Neti, River Charles, Durga Yerasuri

**Affiliations:** Department of Internal Medicine, Wayne State University and Trinity Health Oakland, Pontiac, MI 48341, USA; Wayne State University School of Medicine, Detroit, MI 48201, USA; Wayne State University School of Medicine, Detroit, MI 48201, USA; Wayne State University School of Medicine, Detroit, MI 48201, USA; Arkana Laboratories, Little Rock, AR 72211, USA; Department of Endocrinology, Trinity Health Livonia, Livonia, MI 48154, USA

**Keywords:** renal sarcoidosis, hypercalcemia, acute kidney injury, interdisciplinary collaboration

## Abstract

Renal sarcoidosis is an uncommon extrapulmonary manifestation, rarely presenting with severe hypercalcemia. We describe a diagnostically challenging case of a 56-year-old female with goblet cell adenocarcinoma of the appendix, evaluated for acute kidney injury (AKI) and hypercalcemia after abnormal labs at a routine oncology visit. Initial assessment suggested a parathyroid-independent etiology. Extensive malignancy workup, including imaging and tumor markers, failed to identify an oncologic process. Despite aggressive resuscitation and bisphosphonate therapy, recurrent hypercalcemia with AKI persisted. Low-normal 1,25-dihydroxyvitamin D, and suppressed PTH further complicated diagnosis. Definitive diagnosis was made via renal biopsy, revealing granulomatous interstitial nephritis consistent with renal sarcoidosis. Treatment with corticosteroids led to rapid improvement in calcium and renal function. This case highlights the diagnostic complexity of renal sarcoidosis, especially when presenting with hypercalcemia without systemic clues. The delayed diagnosis underscores the importance of a broad differential and interdisciplinary collaboration in evaluating unexplained hypercalcemia.

## Introduction

Sarcoidosis is a multisystem inflammatory disorder characterized by noncaseating granulomas, most commonly affecting the lungs and lymphatic system. Pulmonary involvement occurs in over 90% of cases, but extrapulmonary manifestations can affect the eyes, heart, nervous system, and kidneys [1]. Renal sarcoidosis is rare, with a reported prevalence of 0.7% to 4.3% among patients with systemic sarcoidosis, typically presenting as interstitial nephritis, nephrocalcinosis, or hypercalcemia—often due to excess 1,25-dihydroxyvitamin D produced by granuloma-activated macrophages [2]. This hypercalcemia can worsen renal function and complicate management.

Hypercalcemia, seen in 10% to 20% of sarcoidosis cases, is rarely the primary presentation [3]. It results from unregulated extrarenal calcitriol production, leading to increased calcium absorption and decreased excretion [4]. Unlike primary hyperparathyroidism, where PTH regulation provides feedback inhibition, sarcoid-related calcitriol production lacks this control, causing persistent hypercalcemia. Chronic hypercalcemia can cause nephrocalcinosis, nephrolithiasis, or granulomatous kidney infiltration, potentially leading to acute kidney injury (AKI) and chronic kidney disease (CKD) [5]. Renal sarcoidosis without systemic involvement, particularly with severe hypercalcemia, is exceedingly rare.

More common causes like primary hyperparathyroidism, malignancy, and multiple myeloma are usually considered first, delaying diagnosis [6]. We present a rare case of renal sarcoidosis with low-normal active vitamin D levels, posing a significant diagnostic challenge.

## Case Presentation

A 56-year-old female with a history of appendiceal goblet cell adenocarcinoma (treated with hemicolectomy and chemotherapy), CKD stage IV, osteoporosis, chronic diarrhea, recurrent AKI, and malnutrition presented with repeated episodes of severe hypercalcemia and AKI. Hypercalcemia was first identified during routine oncology follow-up, leading to multiple hospitalizations and treatment with IV hydration, bisphosphonates, calcitonin, and denosumab. Despite therapy, hypercalcemia persisted, complicating her care and impacting her physical and emotional well-being.

A persistently low-normal 1,25-dihydroxyvitamin D, normal angiotensin-converting enzyme (ACE) level of 32 U/L (≈533 nkat/L; reference range, 8-32 U/L [133-533 nkat/L]) and suppressed PTH created a diagnostic paradox.

**Figure 1.**
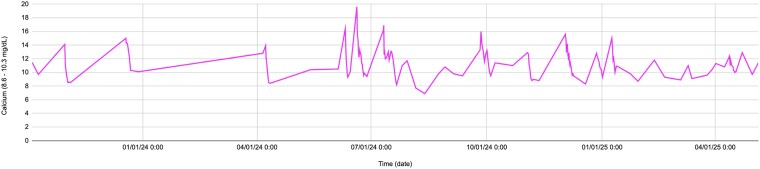
Serum calcium trends. Serum calcium levels over time, with prednisone initiation (black arrow) followed by stabilization, supporting sarcoidosis-related hypercalcemia.

Initial labs showed calcium at 15.0 mg/dL (SI: 3.75 mmol/L) (reference range, 8.5-10.5 mg/dL [SI: 2.12-2.62 mmol/L]) (Fig. 1), corrected calcium 14.2 mg/dL (SI: 3.55 mmol/L), creatinine 2.01 mg/dL [International System of Units (SI): 177.8 µmol/L] (baseline <1.0 mg/dL [SI: < 88.4 µmol/L]), and estimated glomerular filtration rate 29 mL/min/1.73 m² (reference range, > 90 mL/min/1.73 m²). Calcium remained elevated (11.0-15.0 mg/dL [SI: 2.75-3.75 mmol/L]) despite treatment. Retrospective review showed intermittent hypercalcemia for nearly 2 years, peaking at 19.6 mg/dL (SI: 4.90 mmol/L). A comprehensive overview of the patient's laboratory results is detailed in Table 1. She was hospitalized multiple times, receiving pamidronate, zoledronic acid, calcitonin, and denosumab. Hypercalcemia recurred within weeks of treatment, with no definitive diagnosis. At the time of evaluation, her medications included fluoxetine, ropinirole, oxybutynin, hydrocodone-acetaminophen as needed, a multivitamin, potassium chloride (oral and IV formulations), psyllium, and cyanocobalamin.

**Table 1.** Laboratory values at initial presentation

Test	Reference range (conventional)	Patient value (conventional)	Reference range (SI)	Patient value (SI)
Sodium	135-144 mmol/L	136 mmol/L	—	—
Potassium	3.5-5.3 mmol/L	4.0 mmol/L	—	—
Chloride	98-107 mmol/L	91 (L) mmol/L	—	—
Bicarbonate (HCO₃)	21-31 mmol/L	39 (H) mmol/L	—	—
Anion gap	3-11	6	—	—
Glucose	70-99 mg/dL	132 (H) mg/dL	3.9-5.5 mmol/L	7.3 mmol/L
Blood urea nitrogen	7-25 mg/dL	13 mg/dL	2.5-8.9 mmol/L	4.6 mmol/L
Creatinine	0.60-1.20 mg/dL	2.01 (H) mg/dL	53-106 µmol/L	177.5 µmol/L
Estimated glomerular filtration rate	≥60 mL/min/1.73 m²	29 (L) mL	≥1.0 mL/s/1.73 m²	0.48 (L)
Calcium (total)	8.6-10.3 mg/dL	15.0 (H) mg/dL	2.15-2.57 mmol/L	3.74 mmol/L
Corrected calcium	8.6-10.3 mg/dL	14.2 (H) mg/dL	2.15-2.57 mmol/L	3.54 mmol/L
Ionized calcium	4.60-5.40 mg/dL	7.31 (H) mg/dL	1.15-1.35 mmol/L	1.82 mmol/L
Phosphorus	2.4-4.6 mg/dL	3.5 mg/dL	0.78-1.49 mmol/L	1.13 mmol/L
Magnesium	1.7-2.5 mg/dL	2.2 mg/dL	0.70-1.00 mmol/L	0.91 mmol/L
Albumin	3.5-5.7 g/dL	4.6 g/dL	35-57 g/L	46 g/L
Total protein	6.1-7.9 g/dL	7.9 g/dL	61-79 g/L	79 g/L
Bilirubin (total)	0.3-1.0 mg/dL	0.6 mg/dL	5.1-17.1 µmol/L	10.3 µmol/L
Aspartate aminotransferase	13-39 U/L	29 U/L	—	—
Alanine aminotransferase	7-52 U/L	15 U/L	—	—
Alkaline phosphatase	27-120 U/L	38 U/L	—	—
White blood cell count	4.0-10.0 K/µL	4.5 K/µL	4.0-10.0 × 10⁹/L	4.5 × 10⁹/L
Red blood cell count	4.20-5.40 M/µL	3.89 (L) M/µL	4.20-5.40 × 10¹²/L	3.89 × 10¹²/L
Hemoglobin	12.0-16.0 g/dL	12.1 g/dL	120-160 g/L	121 g/L
Hematocrit	36-48%	37%	0.36-0.48	0.37
Platelet count	140-450 K/µL	190 K/µL	140-450 × 10⁹/L	190 × 10⁹/L
Angiotensin-converting enzyme	8-32 U/L	32 U/L	133-533 nkat/L	533 nkat/L
PTH	12-88 pg/mL	14.7 pg/mL	1.3-9.3 pmol/L	1.6 pmol/L
PTH-related protein	11-20 pg/mL	16 pg/mL	1.3-2.3 pmol/L	1.9 pmol/L
Vitamin D, 25-hydroxy	≥30 ng/mL	20.8 (L) ng/mL	≥75 nmol/L	51.9 nmol/L
Vitamin D, 1,25-dihydroxy	15-65 pg/mL	<5 (L) pg/mL	39-169 pmol/L	<13 pmol/L

Comprehensive summary of the patient's laboratory results at the time of presentation, with reference ranges provided in conventional units and patient values shown in both conventional and International System of Units (SI). Abnormal values are indicated as high (H) or low (L).

## Diagnostic Assessment

Malignancy workup—including positron emission tomography, computed tomography (CT), magnetic resonance imaging, tumor markers, and bone marrow biopsy— was negative. Standard imaging for sarcoidosis evaluation, including chest CT and chest radiograph, showed no pulmonary abnormalities. Abdominopelvic imaging, including CT and ultrasound, showed no renal abnormalities. Initial concern for cancer recurrence faded with repeated normal results. Nephrology suspected hypercalcemia-induced nephropathy. Endocrinology ruled out primary hyperparathyroidism due to suppressed PTH and low 1,25-dihydroxyvitamin D < 5 pg/mL (SI: < 13 pmol/L) (reference range, 15-65 pg/mL [39-169 pmol/L]). Urology excluded obstruction, and psychiatry was involved due to worsening anxiety and depression from prolonged illness and diagnostic uncertainty.

She experienced ongoing muscle cramps, weakness, nausea, anorexia, weight loss, and fatigue. The prolonged course caused emotional distress and compounded preexisting mental health conditions. Multiple hospitalizations over 2 years revealed worsening renal insufficiency, metabolic alkalosis, and progressive CKD from stage III to IV. Dialysis became necessary, and a renal biopsy was pursued to clarify the cause of renal deterioration, as this is not routinely performed before initiating dialysis unless diagnosis is uncertain. Evaluation for persistent hypercalcemia prioritized malignancy. However, imaging, bone marrow biopsy, ACE levels, chest studies, and hormonal panels (PTH, PTH-related protein, vitamin D metabolites) were unremarkable. Gastroenterology evaluation, including esophagogastroduodenoscopy biopsies, was negative for malignancy or granulomas. Multiple myeloma was excluded with normal serum free light chains, negative serum and urine protein electrophoresis, and benign marrow biopsy. Primary hyperparathyroidism and vitamin D toxicity were ruled out by suppressed PTH and low 25- and 1,25-dihydroxyvitamin D. Granulomatous disease was not initially suspected due to normal ACE and no systemic or pulmonary features.

**Figure 2.**
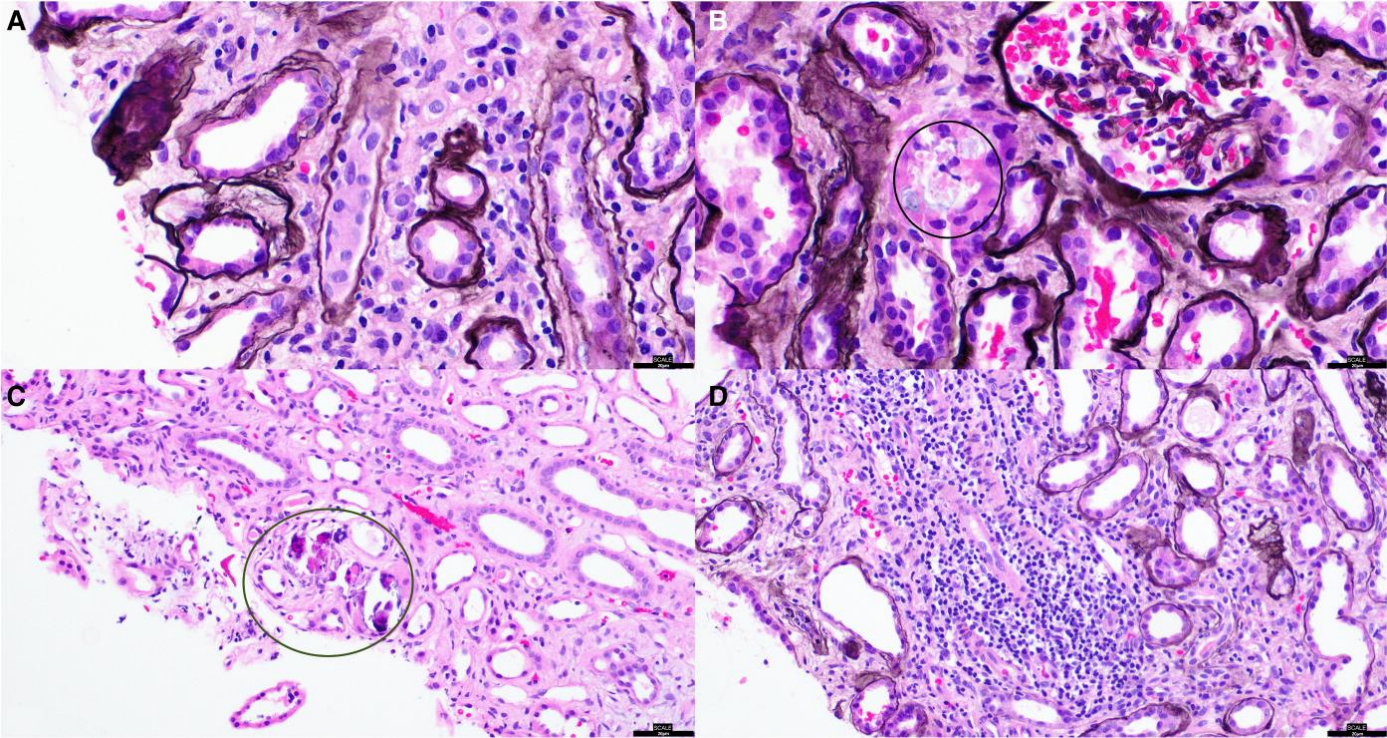
Histopathologic findings from renal biopsy in sarcoidosis. (A) Tubulitis with lymphocytic infiltration of the tubular epithelium. (B) Cortical granuloma with multinucleated giant cell formation. (C) Medullary calcium phosphate deposition consistent with nephrocalcinosis in the setting of chronic hypercalcemia. (D) Dense interstitial inflammation involving the renal parenchyma.

Despite repeated evaluations and standard treatments, hypercalcemia and AKI persisted. Ultimately, a renal biopsy performed after significant decline revealed granulomatous interstitial nephritis, confirming renal sarcoidosis (Fig. 2).

## Treatment

**Figure 3.**
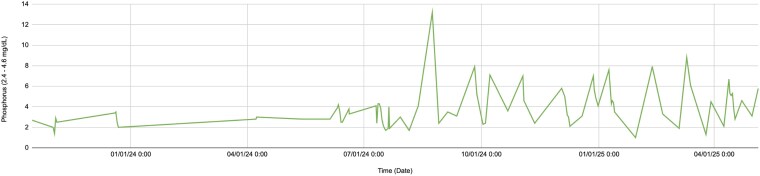
Serum phosphate trends. Phosphate values remained largely within the reference range throughout the disease course.

Following confirmation of renal sarcoidosis, prednisone temporarily normalized calcium levels and improved renal function. However, tapering quickly triggered recurrence, highlighting the challenge of managing sarcoidosis-related hypercalcemia. Interestingly, throughout her disease course, the patient’s phosphate values remained mostly within the reference range (Fig. 3). Due to her osteoporosis, bisphosphonates were cautiously continued under endocrinology oversight. She was advised to stay well hydrated and undergo regular calcium and renal function monitoring.

## Outcome and Follow-up

The patient experienced initial improvement in calcium levels and renal function following IV fluids, bisphosphonate therapy, and corticosteroids. However, due to the complexity of her presentation and the rarity of renal sarcoidosis, she continues to undergo treatment and close follow-up with nephrology, endocrinology, and oncology. Ongoing monitoring is necessary to assess disease progression, response to therapy, and potential complications.

## Discussion

Renal sarcoidosis is a rare but critical manifestation of systemic sarcoidosis, typically presenting as granulomatous interstitial nephritis, nephrocalcinosis, or hypercalcemia. Classically, sarcoid-related hypercalcemia is driven by extrarenal synthesis of 1,25-dihydroxyvitamin D by macrophages within granulomas, leading to increased calcium absorption. However, our patient uniquely exhibited persistently low-normal 1,25-dihydroxyvitamin D levels, complicating the diagnostic process. Severe hypercalcemia in this context is rare and sparsely documented.

Several mechanisms may explain such atypical findings. Advanced renal dysfunction may impair both 1-α-hydroxylation and clearance of vitamin D, distorting typical lab profiles. Additionally, proinflammatory cytokines, especially interleukin-6, may independently promote osteoclast activation and bone resorption, contributing to hypercalcemia without elevated vitamin D metabolites.

A defining feature of this case was the prolonged diagnostic delay despite multispecialty involvement. Suppressed PTH, low-normal vitamin D levels, and negative imaging obscured the diagnosis. Initial concern for malignancy was reasonable given the patient's cancer history, but ongoing diagnostic challenges highlight the limitations of standard hypercalcemia workups. Prior reports have described similar misdiagnoses, with extensive oncologic evaluations preceding a correct sarcoidosis diagnosis [7]. Biopsy site selection is crucial in such cases—renal involvement is rare compared to pulmonary or nodal disease—yet ultimately proved essential in this case.

A report by Mulkareddy et al described a 60-year-old man with hypercalcemia as an initial manifestation of sarcoidosis, identified only after extensive workup [8]. Unlike our case, that patient had elevated vitamin D levels and a positive lymph node biopsy, aiding diagnosis. In contrast, our patient's normal ACE levels and absence of lymphadenopathy prolonged diagnostic uncertainty. This comparison underscores how variability in biomarkers and a lack of systemic signs can obscure the diagnosis of sarcoidosis. Furthermore, Sharma et al described a 61-year-old woman with hypercalcemia, anemia, and AKI later diagnosed with sarcoidosis via bone marrow biopsy [9]. As with our patient, her hypercalcemia was resistant to conventional therapy, raising concern for hematologic malignancy. However, abnormal pulmonary imaging and elevated ACE levels eventually led to a diagnosis—features our patient lacked. This further illustrates the challenge of diagnosing renal sarcoidosis as an isolated entity.

Delayed diagnosis also had consequences for the patient's mental health. Although she denied cognitive changes, the recurring hospitalizations and uncertainty caused clear emotional distress. Studies show increased anxiety and depression among patients with chronic, unexplained conditions, particularly those worsened by misdiagnosis [10]. The complexity of this case highlights the need for interdisciplinary collaboration to ensure rare conditions like renal sarcoidosis are not overlooked.

Treatment was also complicated by the patient's declining renal function. While bisphosphonates and denosumab helped lower calcium levels, they posed additional renal risks. Corticosteroids brought initial improvement, but relapse occurred with tapering—reflecting the refractory nature of sarcoidosis-related hypercalcemia.

In conclusion, this case highlights a diagnostically challenging presentation of renal sarcoidosis, with recurrent hypercalcemia despite low-normal vitamin D levels. It emphasizes the importance of considering renal sarcoidosis early in unexplained hypercalcemia, especially when traditional markers and imaging are inconclusive. Prompt recognition is key to minimizing delays and improving outcomes in complex, rare presentations.

## Learning Points

- Renal sarcoidosis can present with isolated, severe hypercalcemia and AKI, even in the absence of systemic or pulmonary involvement.
- Low or normal 1,25-dihydroxyvitamin D levels do not exclude sarcoidosis-related hypercalcemia and may occur due to impaired renal function or alternative inflammatory pathways.
- A renal biopsy may be considered after thorough noninvasive evaluation if unexplained hypercalcemia with worsening kidney function persists and standard workups remain inconclusive.

## Data Availability

Data sharing is not applicable to this article as no datasets were generated or analyzed during the current study.
